# Analysis of Residual Dependencies of Independent Components Extracted from fMRI Data

**DOI:** 10.1155/2016/2961727

**Published:** 2015-12-29

**Authors:** N. Vanello, E. Ricciardi, L. Landini

**Affiliations:** ^1^Dipartimento di Ingegneria dell'Informazione, University of Pisa, 56122 Pisa, Italy; ^2^Laboratory of Clinical Biochemistry, Department of Experimental Pathology, University of Pisa Medical School, 56126 Pisa, Italy; ^3^Fondazione Toscana Gabriele Monasterio, 56124 Pisa, Italy

## Abstract

Independent component analysis (ICA) of functional magnetic resonance imaging (fMRI) data can be employed as an exploratory method. The lack in the ICA model of strong a priori assumptions about the signal or about the noise leads to difficult interpretations of the results. Moreover, the statistical independence of the components is only approximated. Residual dependencies among the components can reveal informative structure in the data. A major problem is related to model order selection, that is, the number of components to be extracted. Specifically, overestimation may lead to component splitting. In this work, a method based on hierarchical clustering of ICA applied to fMRI datasets is investigated. The clustering algorithm uses a metric based on the mutual information between the ICs. To estimate the similarity measure, a histogram-based technique and one based on kernel density estimation are tested on simulated datasets. Simulations results indicate that the method could be used to cluster components related to the same task and resulting from a splitting process occurring at different model orders. Different performances of the similarity measures were found and discussed. Preliminary results on real data are reported and show that the method can group task related and transiently task related components.

## 1. Introduction

Functional magnetic resonance imaging (fMRI) is a widespread and well-established technique for the in vivo functional exploration of the brain. fMRI data analysis methods can be roughly classified as* confirmatory* or hypothesis-driven methods and* exploratory* or data-driven methods [1–3]. The former are used in order to test the validity of the experimenters' hypotheses but do not allow the detection of unexpected phenomena, that is, effects that are not modelled a priori. On the other hand, data-driven methods provide results that are based on general assumptions about signal generation but are often difficult to be fully interpreted [4].

Independent component analysis (ICA) is one of the most used exploratory methods both for task-associated neural responses and for resting state signal processing and is based on the assumption of statistical independence of the components to be extracted [4]. This method has proven its capabilities of separating physiological components of different origins (e.g., vascular and respiratory), of detecting unexpected phenomena, such as activations transiently time-locked with the stimulus, and of isolating artefact-related signal changes, such as those due to head movements [5, 6].

Nonetheless, ICA approaches present different drawbacks. In fact, the extracted components are difficult to classify since they do not have an explicit order or relationship among themselves [7]. The stochastic nature of the algorithm, the finite number of the observations, and the presence of noise influence the solution reliability and stability. For this reason several approaches have been proposed to face this issue [8–10]. In [8] the reliability of the solution is investigated by clustering the results obtained with multiple runs. A different approach was proposed for solving the same issue in [9] where an alignment of different maps obtained from multiple runs of the algorithm is obtained using information criteria and then the reliability of the components is estimated looking at the consistency and variability with *t* tests. In [10], the independent components are classified according to different measures, as maps dipolarity, which is thought to be related to physiological plausibility of the component, and a consistency measure obtained through the use of surrogate decomposition.

Another issue affects the estimation of ICA model. In fact, the best number of components to be extracted, that is, the model order, is not known a priori. Underestimating the model order may cause an information loss, while overestimating it may produce spurious results or the splitting of interesting components into more components [7, 11]. In [11], the effects of varying model order are investigated in simulated datasets. Noise and signal intensity values as well as dataset dimensions were highlighted as parameters largely influencing this choice. Moreover, the authors suggested that adopting a high model order might lead to different conclusions in the case of group analysis with respect to single subject analysis. Specifically, in the case of intersubject variability of the maps, increasing model order might lead to results difficult to be interpreted, while in single subject analysis a higher model order was seen as a possibility to distinguish subnetworks characterized by a different temporal behavior. In [12], the effects of increasing model orders in a probabilistic group ICA are highlighted. While the stability of ICA decomposition was found to decrease with increasing model order, the use of higher model orders allowed detection of interesting neuroanatomical and functional components. To overcome order indeterminacy problem, various methods have been proposed [13–18]. Specifically, information theoretic criteria, such as Akaike Information Criterion, Minimum Description Length, and Bayesian Information Criterion, have been applied to solve this issue in fMRI data [13–16]. In [13], the classical information theoretic criteria were adapted to account for temporal and spatial correlations in fMRI data using a subsampling technique to obtain independent and identical distributed samples. In [16], the model order selection was obtained by introducing a noisy ICA model within a Bayesian framework [17]. In [18], bootstrap stability technique as applied to PCA reduction step is proposed. At this time, however, there is no general agreement about the best approach to choose ICA model order.

Apart from stability and model order indeterminacy, the interpretation of ICA results must take into account the fact that the statistical independence among the estimated ICs is only approximated by available algorithms. In fact, the presence of noise and the finite number of observations for each measurement do not allow accurate estimation of the higher order statistics [19] used to search for statistical independence. Thus, residual dependence between the extracted components can still be found and can be used to reveal some structure in the dataset. A topographic approach was suggested for ICA [20], where the ICA model was modified to take into account a topographic order among the extracted components: the distance between two elements in the topographic maps is related to the residual dependency. In [21], a comparison of ICA approaches that exploit clustering techniques to estimate the model is performed. Specifically, the topographic approaches are compared with standard ones, as well as with an approach defining a tree-like dependency structure among the components [22]. This work demonstrated that the information inherent in the dependencies among the components can separate artifacts and task related components as well as detecting interesting relationships among components. In a work by Ma et al. [23], the residual dependencies among components were estimated using a similarity measure based on Mutual Information (MI) [24] between the components estimated using a kernel density estimation (KDE) approach [25]. Afterwards, the components are classified using a hierarchical clustering technique [26]. This latter work showed that this approach could group interesting components, as applied to group fMRI data acquired during rest, across different algorithms and model orders. Specifically, it is claimed that the higher dimensionality of clustered components at higher orders is related to the merging of split components.

In this work, we propose an approach similar to the one developed by Ma et al. [23] which differentiates for a number of methodological aspects. Firstly, we explored two different methods for MI estimation, specifically a histogram-based technique and the KDE approach. Moreover, we adopted a different MI-based measure that is a metric in a mathematical sense and a different criterion for the clustering of the algorithm results. Finally, in [23], the robustness of the approach against model order indeterminacy was deduced from the analysis results of real resting state fMRI data. Differently, here we performed an analysis on the ICs estimated from simulated dataset with increasing model orders. By the analysis of the results from simulated and real dataset, we investigated the possibility of improving the grouping of the components originated from a splitting process due to model order overestimation.

## 2. Theory and Methodology

Datasets from fMRI are formed by time sequences of images or volumes, acquired while a subject is performing some sensory/motor or cognitive task at rest. In this work we focused on single subject fMRI data acquired during the execution of a block design task, alternating in time two different conditions. Synthetic data were simulated accordingly.

### 2.1. Simulated Data

A synthetic brain dataset was used for our preliminary studies of the clustering method [27]. Signal increase in response to neural activation was simulated convolving the time course describing the task with a typical hemodynamic response function: we chose the three-parameter gamma variate function defined as *h*(*t*) = *kt* ^8.60^ *e* ^−*t*/0.547^ *u*(*t*) [28], where *k* is a constant proportional to the amplitude of the signal increase. The task description was modeled as a square wave alternating in time: 15 seconds ON with 15 seconds OFF conditions. The total time length of each simulated dataset was three minutes, for a total number of scans equal to 60 (simulated TR = 3 s). The activated regions were created modulating the baseline intensities of a group of voxels, selected using a mask, with the time series previously described. Activated regions are supposed to be limited in a region of connected voxels and were created using a mask formed by region smoothed with a Gaussian kernel to simulate the spatial point spread function due to the vasculature. The Gaussian kernel was a bidimensional one with 3 mm full width half maximum (FWHM) parameter. All the simulations that we present in this paper were obtained by defining two nonoverlapping activated regions of about 2 centimeters of diameter. In the following, these activated regions will be indicated as region of interest (ROI) number 1 and number 2. The signal change was about 2 percent as compared to the baseline level in the center of the activated regions. Different time delays between the activation time courses of the two regions were simulated: 1.25, 2.5, and 5 seconds.

The noise in the images was taken to be i.i.d. Gaussian distributed with zero mean and variance *σ* ^2^. For each delay value, different noise levels were simulated. The simulated noise standard deviation was 0.33%, 0.66%, 1%, and 1.33% of the mean image value at the baseline level. The contrast to noise ratio, defined as CNR = Δ*S*/*σ*, where Δ*S* is the signal change following an activation, was equal to, approximately, 6, 3, 2, and 1.5 (resp.): this was the maximum value at the center of the activated regions.

### 2.2. Real Data

Brain activity was measured in a 25-year-old right-handed healthy female. The subject signed a written informed consent prior to the enrolment into the study under a protocol approved by the Ethics Committee of the University of Pisa, Italy. The scanner used was a 1.5 Tesla, GE Signa Cv/i. An anatomical image was acquired with a 3D SPGR sequence. The functional scans were gradient echo-EPI with TR = 3 sec, TE = 40 msec, and FA = 90 degrees with bandwidth of 62.5 kHz. Twenty axial slices, covering the entire brain, were acquired with slice thickness of 5 mm, 240 mm FOV, and in plane 64 × 64 spatial matrix, and the number of time scans was 63, for a total acquisition time of 189 s. The subject head was restrained with foam in order to minimize head movements. The subject performed a simple finger tapping sequence with her right hand fingers: the task was a block design paradigm alternating 15 sec ON and 15 seconds OFF conditions. The number of scans was 60 for a total run length of three minutes. The images time series were interpolated to correct for slice timing effects and volume registered to a reference scan to reduce movement related effects. The images were then spatially transformed to the Talairach-Tournoux Atlas reference system [29]. These preprocessing steps were performed using AFNI [30].

### 2.3. ICA Model

In spatial ICA we can write the observed data as **x**(*ν*) = [*x*_1_(*ν*), *x*_2_(*ν*),…, *x*_*n*_(*ν*)]^*T*^, where *x*_*i*_ is the *i*th image or volume in the sequence and *ν* is a spatial index for each volume element (voxel). The observed data **x** can be written as a linear mixing of spatial ICs *s*_*i*_:

(1) $$\begin{array}{c} x\left(\;\nu \;\right)=As\left(\;\nu \;\right), \end{array}$$

where **s**(*ν*) = [*s*_1_(*ν*), *s*_2_(*ν*),…, *s*_*n*_(*ν*)]^*T*^. Both *s*_*i*_ and the mixing matrix **A** are unknown. In this model *x*_*i*_ and *s*_*i*_ are seen as random variables and *ν* is an index for the observations of each random variable.

The ICA problem consists in finding an unmixing matrix **W** so that the estimated independent components can be written as $\tilde{s}=Wx$. Under the hypothesis that the mixing matrix is invertible, **W**≅**A** ^−1^, each estimated component ${\tilde{s}}_{i}$ can be written as a linear combination of the observed variables ${\tilde{s}}_{i}=w_{i}^{T}x$, with *w*_*i*_ the *i*th column of **W** and $x=W^{-1}\tilde{s}$. Each ${\tilde{s}}_{i}$ can be seen as a spatial map, individuating a value for every voxel. The *i*th spatial fixed map is time modulated by the corresponding time course, given by the *i*th column of **W** ^−1^. The components, whose associated time courses highly correlate with the paradigm, are considered as task related components or consistently task related (CTR). The components, whose activation is related only partially with the paradigm, are called transiently task related (TTR) components [5]. Several approaches to estimate the ICA have been described (see [6] and references therein) and for the reasons highlighted in the introduction they all lead to components that are only approximately statistically independent. One way to estimate the ICs is a method based on the maximization of the non-Gaussianity of the ${\tilde{s}}_{i}$ [31].

A fast fixed point algorithm [32] can be used to find the weights *w*_*i*_ such that non-Gaussianity of ${\tilde{s}}_{i}$ is maximized. The fastICA algorithm can exploit different nonlinear functions to approximate negentropy whose maximization leads to non-Gaussianity maximization.

In this work the ICA decomposition was obtained using the fastICA algorithm with tanh as nonlinearity [33]. Different model orders were applied, specifically 5, 10, 15, and 20. After the extraction step, each independent map was transformed into *z* map statistics to find the voxels contributing significantly to the corresponding component. Given an independent map *s*_*i*_, the *z* map can be computed as *z* = (*s*_*i*_ − *m*_*i*_)/*σ*_*i*_, where *m*_*i*_ is the mean of the values of *s*_*i*_ and *σ*_*i*_ are their standard deviation.

### 2.4. Proposed Method

We hypothesized that the components resulting from a splitting process due to a model order overestimation show a higher residual dependence with respect to other components. Under this hypothesis, the residual dependencies among the components could be explored to detect split components. We proposed to estimate the residual dependencies using pairwise distance measure between two components, *s*_*i*_ and *s*_*j*_, based upon the definition of mutual information. A hierarchical clustering approach was then employed to classify and visualize the similarities, in terms of distance measure, between the extracted ICs. The clustering results can be visualized by a dendrogram that highlights the merging of the components due to the similarity criterion.

#### 2.4.1. Similarity Measure

The similarity measure between two components *s*_*i*_ and *s*_*j*_ is defined as follows:

(2) $$\begin{array}{c} D\left(\;s_{i},s_{j}\;\right)=H\left(\;s_{i},s_{j}\;\right)-I\left(\;s_{i},s_{j}\;\right), \end{array}$$

where **H**(*s*_*i*_, *s*_*j*_) is the joint entropy and **I**(*s*_*i*_, *s*_*j*_) is the mutual information between two sources. The choice to use **D**(*s*_*i*_, *s*_*j*_) rather than **I**(*s*_*i*_, *s*_*j*_) is based on the fact that the latter is not a distance metric in the mathematical sense [34]. In this work histogram-based technique and a kernel density estimation approach to compute **D**(*s*_*i*_, *s*_*j*_) were compared.

*(1) Histogram-Based Technique*. The probability that a variable value, that is, independent component, lies in the *k*th interval *a*_*k*_ can be estimated as the frequency of occurrence, so that we can write *p*(*s*_*i*_ ⊂ *a*_*k*_) = *N*_*k*_/*N*, where *N* is the total number of observations for *s*_*i*_ and *N*_*k*_ is the number of times *s*_*i*_ belongs to the *k*th interval. This probability in the following will be referred to as *p*(*a*_*k*_).

The probability that the variable *s*_*i*_ lies in the *h*th interval while the variable *s*_*j*_ lies in the *k*th interval is given by *p*(*s*_*i*_ ⊂ *a*_*h*_, *s*_*j*_ ⊂ *a*_*k*_) = *N*_*hk*_/*N*, where *N*_*hk*_ is the number of times the couple (*s*_*i*_, *s*_*j*_) belongs to the bidimensional bin [*h*, *k*]. This quantity can be written as *p*(*a*_*h*_, *a*_*k*_).

We estimated the joint entropy as

(3) $$\begin{array}{c} H\left(\;s_{i},s_{j}\;\right)=-\overset{N_{h}}{\underset{h=1}{\sum }}\overset{N_{k}}{\underset{k=1}{\sum }}p\left(\;a_{h},b_{k}\;\right)\log\left(\;p\left(\;a_{h},b_{k}\;\right)\;\right), \end{array}$$

while the Mutual Information was computed as

(4) $$\begin{array}{c} I\left(\;s_{i},s_{j}\;\right)=\overset{N_{h}}{\underset{h=1}{\sum }}\overset{N_{k}}{\underset{k=1}{\sum }}p\left(\;a_{h},b_{k}\;\right)\log\left(\;\frac{p\left(a_{h},b_{k}\right)}{p\left(a_{h}\right)p\left(b_{k}\right)}\;\right), \end{array}$$

where *N*_*h*_ and *N*_*k*_ are the number of states, or bins, for variable *s*_*i*_ and *s*_*j*_, respectively.

The number of bins was chosen as *M* = 1 + log_2_⁡(*N*) [35], where *N* is the number of observations, that is, the voxels in the image after masking out of brain voxels. A rank ordering operation was used before performing the histogram-based technique; that is, the data values were replaced by their ranks before the histogram is built. This operation facilitates the computation of mutual information as the distributions of the components are transformed into uniform distributions. Moreover, since we were interested in similarities between two distributions, this approach allows the results not to be strongly affected by the marginal distributions of the components. The correlations between two components are preserved and hence the validity of the results.

*(2) Kernel Density Estimation*. Kernel density estimation (KDE) can be used to estimate mutual information [25]. This method consists in estimating the probability density function (pdf) *p*(*x*) of a variable *x* with a linear combination of kernel functions such that

(5) $$\begin{array}{c} \hat{p}\left(\;x\;\right)=\frac{1}{Nh}\overset{N}{\underset{i=1}{\sum }}K\left(\;\frac{x-x\left(i\right)}{h}\;\right), \end{array}$$

where *x*(*i*) is the *i*th value of the variable and *K*(*x*) is a probability density function itself.

A Gaussian kernel *K*(·) can be used to write the pdf as

(6) $$\begin{array}{c} \hat{p}\left(\;x\;\right)=\frac{1}{\sqrt{2\pi }Nh}\overset{N}{\underset{i=1}{\sum }}\exp\left(\;-\frac{{\left(x-x_{i}\right)}^{2}}{2h^{2}}\;\right). \end{array}$$

The parameter *h* is a smoothing parameter, called bandwidth: small values lead to taking into account finer details that can be not interesting or originate from noise; larger values can hide interesting features in the distributions. For Gaussian distributed variables, with variance *σ* ^2^, the values that minimize the mean square error in estimating the density *p*(*x*) have been found to be *h*_opt_ = 1.06*σN* ^−1/5^ [36].

In the bidimensional case, the bidimensional kernel function is given by

(7) $$\begin{array}{c} \hat{p}\left(\;x,y\;\right)=\frac{1}{2\pi Nh^{2}}\overset{N}{\underset{i=1}{\sum }}\exp\left(\;-\frac{d_{i}{\left(x,y\right)}^{2}}{2h^{2}}\;\right) \end{array}$$

with $d_{i}\left(x,y\right)=\sqrt{{\left(x-x_{i}\right)}^{2}+{\left(y-y_{i}\right)}^{2}}$. The bandwidth *h* is related to the underlying distribution as in the monodimensional case. For Gaussian variables an optimal value has been found to be *h*_opt_ ≈ *σ*(4/(*d*+2))^1/(*d* + 4)^ *N* ^−1/(*d* + 4)^ with *d* = 2 and *σ* is the average marginal standard deviation [37].

Given the probability density functions *p*(*x*), *p*(*y*), and *p*(*x*, *y*) the mutual information and the entropy between two variables *x* and *y* can be estimated as

(8) Ix,y=∫x∫ypx,ylog⁡px,ypxpydx dy,Hx,y=∫x∫ypx,ylog⁡px,ydx dy.

These quantities can be estimated with standard procedures for numerical integration. We adopted adaptive Simpson quadrature approach.

#### 2.4.2. Clustering

The hierarchical clustering approach is based on the Ward method [38]. This method consists in merging every possible cluster pair and choosing the one which minimizes the information loss. To estimate this quantity, the error sum-of-squares (ESS) is used:

(9) $$\begin{array}{c} \mathrm{ESS}\left(\;C_{i}\;\right)=\sum_{x\in C_{i}}{\left(\;x-m_{i}\;\right)}^{2}, \end{array}$$

where *m*_*i*_ is the mean of cluster *C*_*i*_ and *x* are the data points. The distance between two clusters is defined by

(10) dWardC1,C2=ESS⁡C1∪C2−ESSC1−ESSC2.

At each stage, the Ward method merges two groups such that their *d*_Ward_ will be minimized. The merging of the components can be visualized with a dendrogram: the abscissa of the dendrogram indicates the components, while the ordinate level, at which two components or groups of components are merged, is related to the change in the error sum-of-squares after joining groups.

### 2.5. Method Validation

The performance of the proposed method can be evaluated on simulated datasets by verifying that the dendrogram correctly merges the components that were split by the ICA algorithm. To identify the components maps that were related to the simulated activations, receiver operating characteristics (ROC) curves [39] were used and applied to the obtained ICA maps. ROC curves are plots of true positive detection fraction against false positive detection fraction obtained varying the threshold level of the *z* maps. In the case of simulated dataset, the true activated areas are known and serve as ground truth measure, so that it is possible to estimate true positives by looking at the activated voxels, that is, over *z* threshold, in one independent component and by checking whether they correspond to the known activated regions. An area under curve (AUC) estimated from the ROC curve ranging from 0.7 to 0.8 is considered a result showing a fair accuracy of the test performed, whereas an AUC between 0.6 and 0.7 is considered a poor accuracy index [40]. An independent component whose AUC is significant for both ROIs is a component that merges both the two activated regions.

Since we focused on fMRI data acquired during a task execution, the classification of the results can take into account the corresponding time course of each component. Specifically, interesting CTR and TTR components can be highlighted.

## 3. Results

### 3.1. Simulated Datasets

Results from the application of the ICA algorithm on simulated datasets are summarized in Tables 1, 2, and 3. Each table is related to the result obtained from the datasets with time delays between the activations time courses of 1.25, 2.5, and 5 seconds, respectively. For each table different noise levels results are summarized. The two activated regions are indicated by ROIs #1 and #2. In each column, the ICs whose maps result in an AUC greater than 0.6 are shown. Specifically the IC index is shown, in brackets, along with the corresponding AUC.

In the case of the shortest time delay between the activations time courses, with noise standard deviation greater than 0.33%, the two ROIs are not distinguished by the ICA model (see Table 1): so the same IC index appears in both columns. The same results are obtained for a time delay of 2.5 seconds at higher noise levels (see Table 2). For time delay equal to 5 seconds, with the chosen noise levels, the ICA could always distinguish the two activated regions (see Table 3). The model order 5 in most cases allows the separation of each activation in single IC maps. In some cases, using this model order the component splitting occurs. For higher model orders, the splitting process occurs at all noise levels for time delay of 1.25 and 2.5 seconds, while for time delay of 5 s one of the two activated regions is often described by one IC.

**Figure 1:**
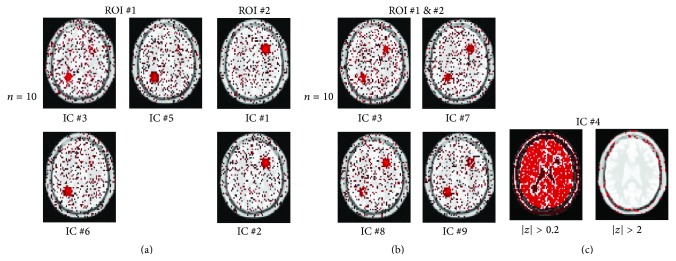
IC maps extracted from simulated datasets. The IC maps (in red-yellow) are transformed in *z*-scores maps, thresholded, and superimposed on the anatomy. Only the ICs related to activated ROI #1 and ROI #2 are shown. (a) Noise level *σ* = 0.66% and time delay is 2.5 s; model order *n* = 10. (b) Model order *n* = 10, noise level *σ* = 1%, and time delay is 1.25 s. In this case, ICA could not separate the two activated regions in different components. Specifically, the activated regions are described by four IC maps. Each map described both ROI #1 and ROI #2. (c) The IC map number 4 estimated in the case of noise level *σ* = 0.66% and time delay of 1.25 seconds with model order = 15. To better show the intensity map distribution this IC is shown twice using two different thresholds applied to the *z*-score statistics: the spatial distribution of the map is flatter with respect to the components related to the ROIs.

In Figure 1(a), the IC maps related to the activated regions, obtained from a simulated dataset with added noise standard deviation *σ* = 0.66% and time delay between the two simulated activations equal to 2.5 s, are shown. The results were obtained for model order *n* = 10. The IC maps, in red-yellow, are transformed in* z*-scores maps, thresholded choosing |*z*| larger than or equal to 2, and superimposed on the anatomy. The two ROIs are described by different ICs and each ROI is described by more than one component. In Figure 1(b), four IC maps extracted from simulated datasets with noise level *σ* = 1% and time delay between simulated activation equal to 1.25 s are shown. In this case the ICA could not separate the two activated regions and the CTR ICs, obtained for model order *n* = 10, are related to both ROIs.

An IC that seems to represent the intensity distribution of all pixels in the images is estimated at the different model orders. The IC has a sub-Gaussian distribution and might originate from the small changes in the baseline mean level, after removing the average value from each image in the sequence (centering operation), as described in Section 2.3. This phenomenon depends on the fact that imposed activations in both ROIs affect the mean level of the image synchronously. As can be seen in Figure 1(c), where a component with the above-described characteristics is shown, the map has some complementary distribution with respect to the activated regions.

#### 3.1.1. Histogram-Based Technique

**Figure 2:**
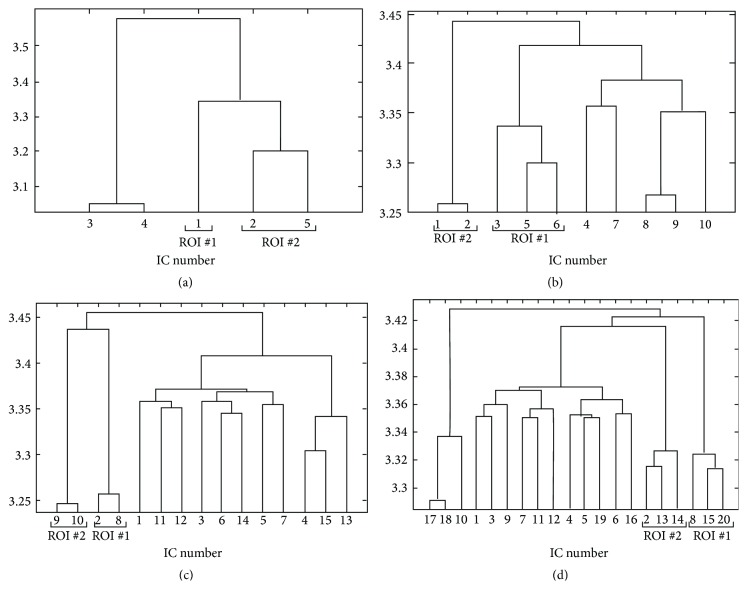
Dendrogram obtained from clustering the pairwise mutual distances between extracted ICs from simulated datasets in the case of noise level *σ* = 0.66% and time delay of 2.5 seconds. The algorithm for estimating the pairwise distances is the histogram-based approach with rank ordering. The results obtained with model order = 5 (a), 10 (b), 15 (c), and 20 (d) are shown.

**Figure 3:**
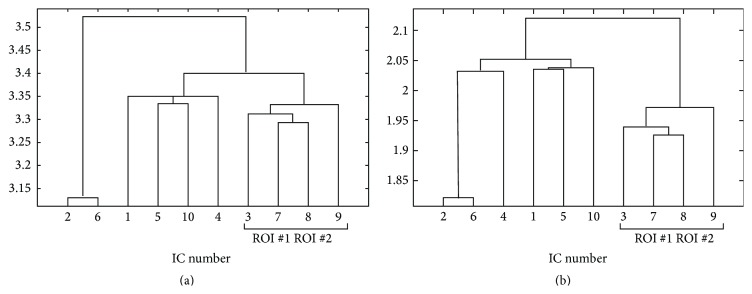
Dendrograms obtained from clustering of the pairwise mutual distances, applied to distances estimated with histogram-based approach (a) and KDE approach (b) between extracted ICs in the case of noise level *σ* = 1% and time delay of 1.25 seconds with model order = 10. The relative IC maps are shown in Figure 1.

The histogram-based technique was applied to ICs extracted from simulated datasets and the pairwise distances were estimated. Since they are representative, only the results for noise level equal to 0.66% and time delay equal to 2.5 seconds are shown in Figure 2. All other cases will be discussed. The hierarchical clustering algorithms successfully merged the components that were found related to the same activated regions. Specifically, in all cases that are listed in Tables 1–3, when the ICA algorithm succeeds in separating the two activated regions, that is, there are no ICs that are related to both ROIs, the clustering algorithm can merge the components that are related to the different ROIs. In Figure 3(a), the results for noise level equal to 1% and time delay of 1.25 seconds are shown: as it can be seen from Table 1, the two activation regions are not distinguished by the ICA model and IC components describing ROI #1 and ROI #2 are merged together. The corresponding IC maps are shown in Figure 1(b).

The ICs related to different activations were often grouped together for model order equal to 5. In all other cases, when ICA detects two separated areas, the two components are grouped in different homogeneous clusters. In Figure 2(a), the components describing the ROI #2 that were separated in two IC components were merged together and then merged again with ROI #1 component. In each case, the components corresponding to the same activation regions are merged together.

#### 3.1.2. Kernel Density Estimation

**Figure 4:**
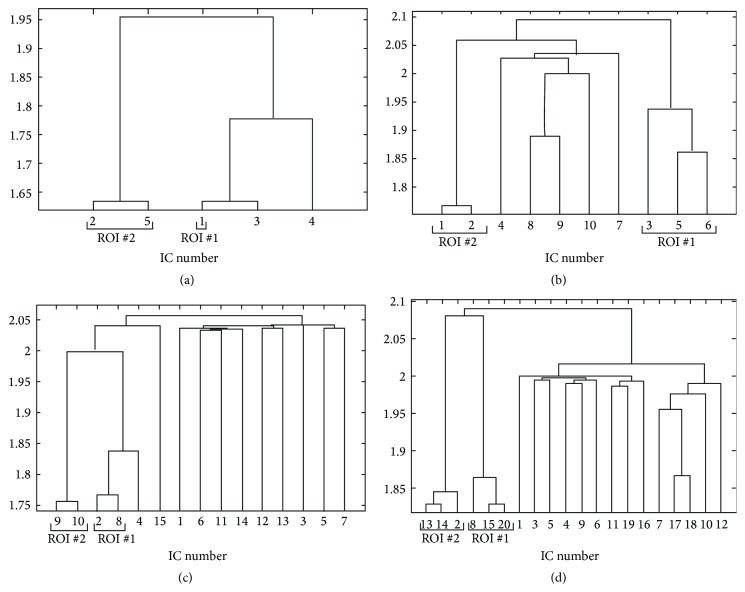
Dendrogram obtained from clustering of the pairwise mutual distances between extracted ICs from simulated datasets in the case of noise level *σ* = 0.66% and time delay of 2.5 seconds. The algorithm for estimating the pairwise distances is the kernel-based one. The results obtained with model order = 5 (a), 10 (b), 15 (c), and 20 (d) are shown.

The analyses described in the previous subsubsection were repeated using the kernel density estimation based method. The results are similar to those obtained for the previous histogram-based approach. Specifically, when the ICA algorithm succeeds in separating the two activated regions the KDE approach can merge the corresponding components in different clusters. As for the histogram-based technique, the components that describe both ROIs simultaneously, occurring when the IC composition could not separate them, are merged together. At the same time, low model orders can result in a merging of the activated regions, albeit found separated by the ICA algorithm. The results pertaining to noise level equal to 0.66% and time delay equal to 2.5 seconds are shown in Figure 4.

#### 3.1.3. Comparison of Histogram-Based and Kernel Density Estimation Approaches

Some differences in the results were found by the two approaches. One difference pertains the ordinates in the dendrograms. The ordinates are related to the increase in within group error sum-of-squares after joining two clusters. In the case of dendrograms corresponding to KDE method the heights of the clusters, that is, the Ward distances, are lower than those obtained from histogram-based technique, meaning a smaller increase in the within-cluster sums of squares after joining the groups in the former case. This fact shows that the distances estimated from the KDE method are on average more homogeneous.

On the other side, the KDE approach was more prone than the histogram-based approach to the clustering of the sub-Gaussian component described in Section 3.1 with a component describing one of the activated regions. This phenomenon can be seen in Figures 4(c) and 4(a). The component #4 is the one shown in Figure 1(c). As a comparison, in Figures 2(c) and 2(a), the results of the histogram-based approach applied to the same dataset are shown.

### 3.2. Real Datasets

In the case of real data, it was not possible to estimate an AUC performance parameter, since we did not know a priori the spatial distribution of the activated regions. It was possible to detect interesting components by looking at the correlation coefficient between the associated time course for each map and the expected hemodynamic time course, given the experimental paradigm. Another criterion that could be used to classify the components in real datasets was related to previous knowledge about the regions involved for the specific task [41]. Moreover, a comparison with the results obtained with a hypothesis driven analysis technique, such as the general linear model (GLM), [1, 42] can be used to detect the regions that are consistently task related and should be included in ICA results.

#### 3.2.1. GLM Results

**Figure 5:**
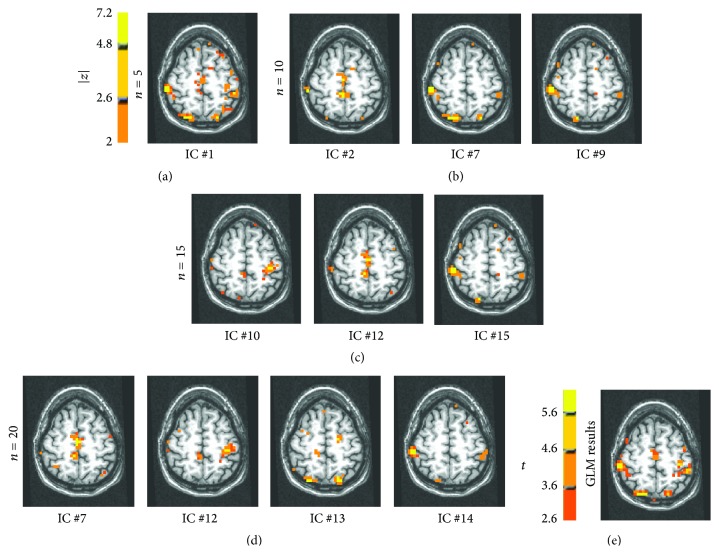
(a)–(d) The IC maps superimposed upon an anatomical T1 weighted image and thresholded with |*z* | > 2 for different model orders are shown. The orientation is RAI. A color bar for the correspondence between color and *z* value is shown. (a) Consistently task related (CTR, *ρ* = 0.84) IC for real dataset with model order equal to 5. (b) ICs maps extracted with model order equal to 10. Component #2 (TTR, *ρ* = 0.43) pertains the SMA and Brodmann area 5 activity. The component number 7 (CTR, *ρ* = 0.74) is related to right and left precuneus, in the primary sensory and motor areas while the component #9 (CTR, *ρ* = 0.9) is related to contralateral primary motor area (the orientation is RAI), SMA, and right precuneus. (c) Resulting ICs from real dataset with model order equal to 15. The component #10 (CTR, *ρ* = 0.86) is mainly related to the contralateral motor area. The activity in the SMA and Brodmann area 5 is mainly described by component #12 (TTR, *ρ* = 0.5). The component #15 (CTR, *ρ* = 0.71) describes the ipsilateral and contralateral sensory areas and right precuneus. (d) CTR ICs for real dataset with model order equal to 20. The component #7 (TTR, *ρ* = 0.59) is related to SMA activity and Brodmann area 5. The component #12 (CTR, *ρ* = 0.73) is mainly related to the contralateral motor area, SMA, and Brodmann area 5 as well. The activity in the precuneus is mainly described by component #13 (CTR, *ρ* = 0.87). The activity of contralateral and ipsilateral sensory areas is described by map #14 (CTR, *ρ* = 0.74). (e) GLM analysis results of the real fMRI dataset. The value of *t* test about the parameter of interest is shown. A color bar related to *t* values is shown.

The results of the GLM applied to the real dataset will be shown. The expected hemodynamic response, used as a regressor of interest, was estimated as the boxcar time series describing the paradigm, convolved with hemodynamic response function [28]. The preprocessing steps for linear analysis are the same as those for ICA. The errors are considered to be normally distributed. The best linear unbiased estimator (BLUE) [43] for the parameter of interest is used, and the null hypothesis of zero activation was tested with *t* test. The results of the GLM applied to real data are shown in Figure 5(e). A *t* statistic map estimated for the coefficient of the regressor of interest of an axial slice at *z* = +52 mm in Talairach-Tournoux Atlas [29] coordinates is superimposed on a T1 weighted anatomical image. The dataset we took into account has an approximated CNR equal to 3, as estimated on the selected slice. The threshold was chosen at *t* = 2.6, corresponding to an uncorrected *p* value lower than 0.01. Significant activations are found in the ipsilateral and contralateral primary motor cortex and in the supplementary motor area (SMA). Right and left precuneus and posterior parietal areas were additionally recruited.

#### 3.2.2. ICA Results

The ICA model was applied to this real dataset with a model order equal to 5, 10, 15, and 20. With a model order equal to 5, one component was detected to be consistently task related (*ρ* = 0.84). In Figure 5(a), the corresponding map (IC #1), thresholded at |*z*| > 2, is shown to be superimposed at an anatomical T1 weighted mask. All the functional areas that were found to be activated using the GLM approach (Figure 5(e)) are found to be described by this IC map. Moreover, a small activation in left superior parietal lobule is described.

Using model order equal to 10, two consistently task related components and a TTR component were found by the ICA. The results are shown in Figure 5(b): the activity in the SMA is depicted mainly by IC #2 (TTR, *ρ* = 0.43). This component shows activation also in Brodmann area 5, not previously found. The component IC #7 (CTR, *ρ* = 0.74) is related to the activity in the precuneus and in the primary sensory and motor areas, while IC #9 (CTR, *ρ* = 0.9) shows activation in the contralateral primary motor area and minor activations in the SMA, Brodmann area 5, and right precuneus. The regions are those found by the ICA with model order = 5 but these seem to be split into several components with model order = 10. On the other side, model order equal to 5 did not detect the activity in Brodmann area 5.

The IC analysis results for model order equal to 15 are shown in Figure 5(c). Two consistently task related areas and one transiently task related area were found. Specifically, component number 15 (CTR, *ρ* = 0.71) describes the ipsilateral and contralateral sensory areas and right precuneus and component number 10 (CTR, *ρ* = 0.86) that is mainly related to the contralateral motor area. The activity in the SMA and Brodmann area 5 is mainly described by a transiently task related component number 12 (TTR, *ρ* = 0.5) shown in the center image.

The IC analysis results for model order equal to 20 are shown in Figure 5(d). Four CTR maps are found: the activity in the right and left precuneus is well described by CTR component number 13 (CTR, *ρ* = 0.73). Activity in SMA is described by CTR component number 12 (CTR, *ρ* = 0.87) and transiently task related component number 7 (TTR, *ρ* = 0.59). While activity in ipsilateral and contralateral motor and sensory areas is described by maps 12 and 14 (CTR, *ρ* = 0.74).

#### 3.2.3. Clustering with Histogram-Based Technique

**Figure 6:**
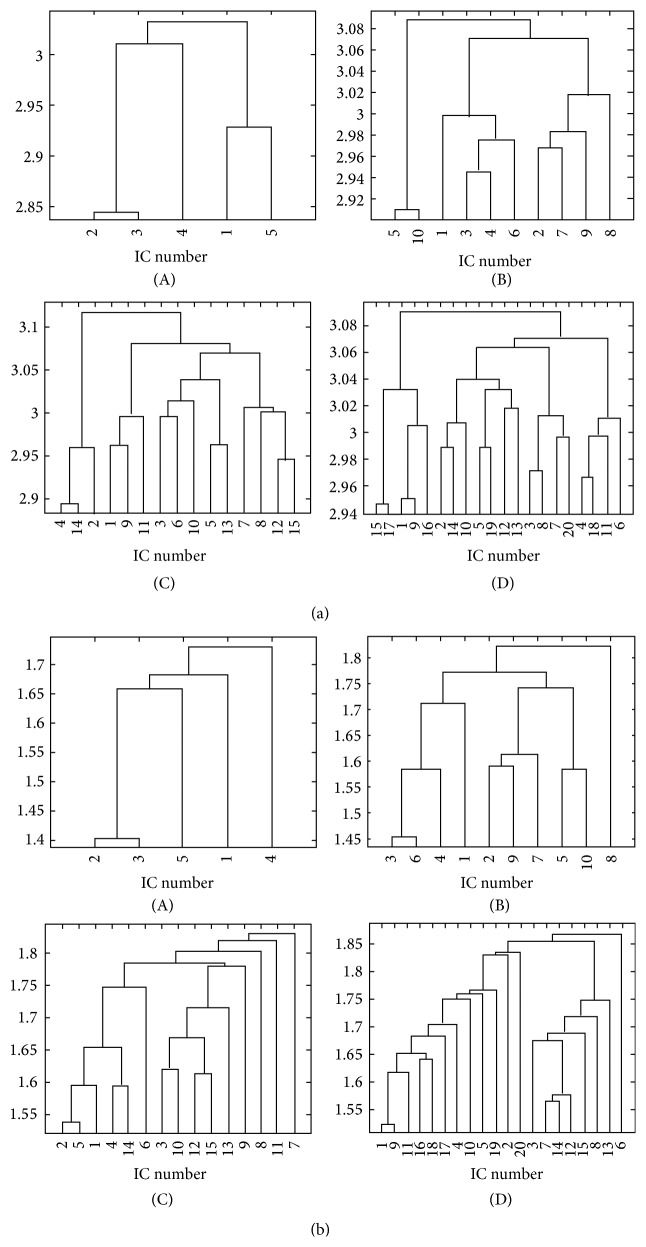
Dendrogram obtained from clustering of the pairwise mutual distances between extracted ICs from real data. The distances were estimated through a histogram-based technique after rank ordering (a) and KDE technique (b). The clustering operation was repeated for different IC model orders: (A) 5, (B) 10, (C) 15, and (D) 20. The interesting CTR components are described in Section 3.2.2. See text for a discussion.

Figure 6(a) depicts the hierarchical clustering algorithm results, applied to distances estimated using the histogram-based technique. In graph (A), the clustering results for model order equal to 5 are shown: the clustering operation in this case was not easy to interpret since only one CTR component was found by ICA (IC #1). For model order equal to 10 (Figure 6(a), graph (B)) the interesting components (ICs #2, #7, and #9) are merged together by the hierarchical clustering algorithm before any other components are merged with these ICs.

**Figure 7:**
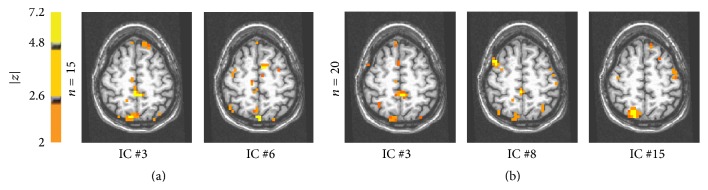
(a) Component maps #3 and #6 obtained from real data, with model order = 15. (b) IC maps extracted from real fMRI data with ICA and model order equal to 20. These maps were merged with CTR components by the clustering algorithm with kernel density estimation. On the left component #3 is shown, in the center component #8 is shown, and on the right component #15 is shown.

Results for model order equal to 15 are shown in graph (C): components number 12 and 15 are merged together. The CTR component number 10 is merged with components number 3 and 6. While component 3 seems to be related to activity in SMA, Brodmann area 5, and right precuneus, component number 6 seems to be related to vascular related signal changes (see Figure 7(a)).

Results for model order equal to 20 are shown in graph (D) of Figure 6(a): only components number 12 and 13 are merged together. It was not possible to identify significant clusters with this model order.

#### 3.2.4. Clustering with KDE Technique

The results of clustering operation obtained on the distances estimated with kernel density approach are shown in Figure 6(b). Graph (A) describes the dendrogram after ICA with model order equal to 5: as for histogram-based technique (see previous subsubsection) there is only one CTR component and no significant clustering of this component is evident. In graph (B), the results for model order equal to 10 are shown: the task related components, #2, #7, and #9, are merged together by the algorithm. The results for model order equal to 15 are shown in graph (C): components #10, #12, and #15 are merged together. Component number 10 is merged with component number 3 whose spatial distribution is shown in Figure 7(a).

In graph (D) of Figure 6(b), the clustering of components after ICA with model order equal to 20 is shown. A cluster of components number #7, #12, and #14 was found. At higher levels components #3, #8, and #15 (Figure 7(b)) and the CTR component #13 were merged. The component number 3 depicts activity in Brodmann area 5 and right precuneus. Component number 15 shows significant activation in right precuneus and left precentral gyrus. Component number 8 shows activation not previously detected in the right middle frontal gyrus and minor activations in Brodmann area 5.

## 4. Discussion

The method proposed here aimed at studying the residual dependencies between the ICs to reveal some informative structure in brain functional data acquired during fMRI protocol. In this work, our aim was twofold: to verify whether this approach could identify and merge the ICs derived from a splitting process caused by an overestimation of ICA model order and to compare two different strategies for the estimation of mutual information-based metric for the analysis of ICs dependencies. Previous work [23] demonstrated that this method can group anatomically and functionally related components from real fMRI datasets acquired during rest. This behavior was found consistently across different model orders. Here, using simulated task related fMRI activations we specifically addressed the issue related to the merging of split components at different model orders with different time relationships between the activated regions and at different contrast to noise ratios.

Moreover, in [23] the mutual information measure adopted to explore the residual dependencies was estimated using the KDE approach. Here, we compared the performances of the latter approach and a histogram-based approach. The histogram-based technique is the classical way to estimate entropy and mutual information [44] and requires that the estimated sources values be partitioned into bins or intervals. An adaptive partitioning operation is used so that the results are not strongly affected by the individual marginal distributions of the components. To achieve this, a rank ordering operation of the elements of each IC is applied [45]. The second method for estimating MI is based on kernel density estimation (KDE) [25]. The KDE method shows a better convergence rate of the MI estimate to the underlying real density than the histogram-based technique and is not sensitive to the choice of the origin. On the other hand, the KDE results are dependent on the choice of kernel functions parameters that are not known a priori.

The ICA of the simulated datasets presented in this paper clearly demonstrated that model order inaccuracy may cause splitting of the same activation region into more components. In this case, the proposed clustering approach highlights the components that have been split. In fact, the clustering algorithm succeeded in grouping together components belonging to the same region of activation found by ICA. Specifically, those components are found to merge earlier in the dendrogram with respect to other components. To classify the independent components that were related to the task we adopted the ROC curves. Specifically, the area under the ROC curve (AUC) was introduced as an index to determine the ROIs to which each component is related. The low values of the AUC parameter were due to the fact that the same activated region is decomposed in different ICs. In some cases the ICA algorithm fails in separating the components. This phenomenon depends upon the time delay between the activation time series and upon the contrast to noise ratio. Even in the cases when the ICA cannot separate the components, as it can be found from dendrogram analysis, the clustering operation correctly associates these components to the same cluster. However, at this time, we cannot claim that this approach could offer the possibility of choosing the right model order of ICA analysis. Nonetheless, exploring the earlier merging of some components may represent the way to reveal brain areas that are related to the same phenomenon of interest. The clustering algorithm with both methods for estimating the MI was found to merge the ICs related to different areas for model order equal to 5. For higher model orders this was never observed. This observation might confirm the need of using higher model order with respect to lower ones [46].

The performances of the hierarchical clustering on simulated datasets, by estimating the pairwise distances with the two approaches, the histogram-based technique and the kernel density estimation one, are different. One difference is related to the homogeneity of the clusters that can be seen looking at the ordinates of the clusters' height. Specifically, in the KDE method, the clusters are more homogeneous with respect to those found by the histogram-based technique. This can be seen by looking at the heights of the clusters that were lower for the former approach. However, with regard to simulated datasets, the KDE approach was more prone than the histogram-based approach to merge a specific sub-Gaussian IC to one of the ICs related to the activated regions. This component may be a result of the mathematical process used to synthesize our data which caused small changes in the baseline mean level of the image voxels. Thus this component might arise as a diffuse activation when the variables centering operation needed for ICA is performed. Nonetheless, the KDE based approach showed the merging of this sub-Gaussian component more frequently than the histogram-based approach, even at higher model orders. Interestingly in [11, 12] larger diffuse, low kurtosis components were found to merge to other components at low model orders, while they were separated at higher orders. This could be further investigated to highlight whether this issue of the KDE approach is relevant when ICA is applied to real fMRI data and whether the choice of a different criterion for the kernel size could alleviate it.

In the case of the real dataset here studied, both methods seem to provide good results for model order equal to ten. For higher order models, the kernel density estimation based method gives better results, merging interesting components both for model order equal to 15 and for model order equal to 20, while the histogram-based technique does not always group interesting components. The ICA could identify activation in areas not found by the GLM as the activity in Brodmann area 5 and left superior parietal lobule. It is interesting to note that Brodmann area 5 was found by ICA with model orders greater than 5: in all cases the hierarchical clustering stage could merge this TTR component with the other CTR.

In this work we did not take into account the stochastic nature of the ICA algorithm and of the noise that could lead to different results on different runs. Nonetheless, consistent results were here obtained across the different 48 simulated datasets obtained by varying the above-mentioned parameters. To face this issue, the results obtained after different executions of the ICA algorithm for several times on the same combinations of noise, time delays, and model order could be done.

Other methodological differences distinguish our work from [23]. In fact, from the methodological point of view, the MI-based metric and the clustering procedures adopted here were different. Future work could evaluate how such different choices influence the final results.

One important issue that was not studied here is the choice of threshold or the dendrogram level used to determine the clusters of interest: while this value could be individuated for simulated datasets, a systematic approach while changing the data volume dimension, the size and distributions of the activation, and the noise level is missing for real data. Moreover, a criterion for the choice of the threshold would require a study with a larger number of participants as well as tasks. Other approaches could be used. In [23], the iterative use of statistical tests was applied to the mean between-clusters and within-cluster distances as a stopping procedure for the dendrogram partitioning.

In this work, we have chosen to apply this method to results obtained from the fastICA algorithm that was proven to work efficiently within the context of fMRI data analysis [47]. However, the proposed method can be applied to results from other ICA algorithms, since it is completely independent from the algorithm employed. With regard to this point, the approach proposed here differs from the one described in [21], in which the ICA algorithms were modified to take into account a topographical order or a dependence structure among the components. Moreover, our goal was not to test the reliability or the stability of the ICA algorithms as in [8–10]. Specifically, the proposed approach could be applied to the maps resulting from the above-cited methods, with the aim of acquiring different information about the relationship among the components. As an example, it could be applied to the centrotypes of the clusters detected by the ICASSO method described in [8].

Another future development of the study presented here could be the analysis and the comparison of the performances of the histogram and KDE based approaches on components extracted at group level, where the intersubject variability represents a relevant issue [11]. Moreover, the evaluation of the proposed approach on simulated data is focused on a specific scenario characterized by two nonoverlapping areas with similar albeit delayed activation time series. Since the fMRI studies have reached a high level of complexity and often are focused on brain activity during rest, different tests should be further developed in the future.

Although limited, the test results presented here indicate that this approach could give fruitful information regarding the statistical similarity of the obtained independent components and cope with the issue of component splitting.

## Figures and Tables

**Table 1:** Evaluation of ICA of the dataset with activations in ROI #1 and ROI #2 with time delay of 1.25 seconds at different noise levels and model orders. The AUC is shown near the IC index (in brackets).

Activations delay, 1.25 sec	Number of ICs	ROI #1 (IC index) AUC	ROI #2 (IC index) AUC
Noise *σ* = 0.33%	5	(1) 0.83	(5) 0.90
	10	(3) 0.77 (6) 0.71	(4) 0.72 (5) 0.77
	15	(4) 0.77 (14) 0.77	(1) 0.79 (3) 0.77
	20	(4) 0.76 (13) 0.78	(5) 0.74 (6) 0.69 (9) 0.77

Noise *σ* = 0.66%	5	(2) 0.68 (5) 0.8	(2) 0.78 (5) 0.7
	10	(4) 0.72 (7) 0.72 (8) 0.7	(4) 0.68 (6) 0.72 (7) 0.72
	15	(2) 0.7 (8) 0.75	(4) 0.75 (7) 0.68
	20	(1) 0.71 (11) 0.75 (20) 0.66	(10) 0.73 (11) 0.71 (20) 0.7

Noise *σ* = 1.00%	5	(4) 0.78	(4) 0.78
	10	(3) 0.7 (7) 0.68 (8) 0.62 (9) 0.67	(3) 0.7 (7) 0.7 (8) 0.7 (9) 0.65
	15	(5) 0.77 (6) 0.77 (12) 0.74 (13) 0.68	(5) 0.75 (6) 0.75 (12) 0.75 (13) 0.73
	20	(18) 0.7 (20) 0.73	(18) 0.73 (20) 0.74

Noise *σ* = 1.33%	5	(1) 0.77	(1) 0.79
	10	(2) 0.73 (3) 0.74	(2) 0.7 (3) 0.76
	15	(8) 0.75 (11) 0.67	(8) 0.71 (11) 0.72
	20	(4) 0.72 (20) 0.66	(4) 0.7 (20) 0.73

**Table 2:** Evaluation of ICA of the dataset with activations in ROI #1 and ROI #2 with time delay of 2.5 seconds at different noise levels and model orders. The AUC is shown near the IC index (in brackets).

Activations delay, 2.5 sec	Number of ICs	ROI #1 (IC index) AUC	ROI #2 (IC index) AUC
Noise *σ* = 0.33%	5	(2) 0.91	(3) 0.89
	10	(3) 0.78 (8) 0.8	(1) 0.76 (2) 0.74 (9) 0.77
	15	(5) 0.8 (7) 0.8	(9) 0.8 (10) 0.8 (11) 0.71
	20	(2) 0.7 (5) 0.7 (6) 0.7 (15) 0.74	(11) 0.77 (17) 0.79 (19) 0.77

Noise *σ* = 0.66%	5	(1) 0.82	(2) 0.78 (5) 0.79
	10	(3) 0.67 (5) 0.79 (6) 0.76	(1) 0.78 (2) 0.77
	15	(2) 0.77 (8) 0.78	(10) 0.78 (9) 0.8
	20	(8) 0.74 (15) 0.77 (20) 0.72	(2) 0.73 (13) 0.78 (14) 0.77

Noise *σ* = 1.00%	5	(1) 0.71 (2) 0.77	(1) 0.82
	10	(1) 0.75 (5) 0.8 (7) 0.69	(4) 0.75 (10) 0.76
	15	(1) 0.73 (3) 0.7	(6) 0.7 (14) 0.74 (15) 0.72
	20	(2) 0.68 (6) 0.74	(2) 0.7 (17) 0.79

Noise *σ* = 1.33%	5	(3) 0.75 (4) 0.67	(3) 0.68 (4) 0.75
	10	(4) 0.65 (10) 0. 71	(4) 0.76 (10) 0.61
	15	(11) 0.74 (14) 0.65	(11) 0.65 (14) 0.74
	20	(4) 0.74 (16) 0.64	(6) 0.69 (16) 0.67

**Table 3:** Evaluation of ICA of the dataset with activations in ROI #1 and ROI #2 with time delay of 5 seconds at different noise levels and model orders. The AUC is shown near the IC index (in brackets).

Activations delay, 5 sec	Number of ICs	ROI #1 (IC index) AUC	ROI #2 (IC index) AUC
Noise *σ* = 0.33%	5	(5) 0.9	(1) 0.9
	10	(3) 0.77 (7) 0.76 (8) 0.78	(5) 0.76 (9) 0.79 (10) 0.76
	15	(6) 0.79 (10) 0.74 (14) 0.78	(4) 0.74 (11) 0.8
	20	(19) 0.9	(8) 0.74 (11) 0.76 (17) 0.77

Noise *σ* = 0.66%	5	(4) 0.83	(1) 0.85
	10	(2) 0.72 (3) 0.77	(5) 0.84
	15	(7) 0.74 (12) 0.76	(2) 0.75 (4) 0.74
	20	(2) 0.76 (3) 0.75	(11) 0.78 (16) 0.75

Noise *σ* = 1.00%	5	(1) 0.81	(5) 0.83
	10	(9) 0.74 (10) 0.77	(3) 0.76 (7) 0.76
	15	(5) 0.7 (10) 0.68 (15) 0.73	(1) 0.76 (8) 0.73 (11) 0.7
	20	(15) 0.78 (16) 0.72	(1) 0.77 (14) 0.80

Noise *σ* = 1.33%	5	(4) 0.84	(5) 0.8
	10	(9) 0.82	(4) 0.76 (5) 0.76
	15	(14) 0.83	(5) 0.71 (11) 0.76
	20	(12) 0.85	(2) 0.76 (15) 0.76
